# 1,2,4,5-Tetra­fluoro-3,6-diiodo­benzene–2,3-bis­(pyridin-2-yl)pyrazine (1/1)

**DOI:** 10.1107/S1600536810041668

**Published:** 2010-10-23

**Authors:** Hadi D. Arman, Trupta Kaulgud, Edward R. T. Tiekink

**Affiliations:** a Department of Chemistry, The University of Texas at San Antonio, One UTSA Circle, San Antonio, Texas 78249-0698, USA; b Department of Chemistry, University of Malaya, 50603 Kuala Lumpur, Malaysia

## Abstract

The components of the title 1:1 co-crystal, C_14_H_10_N_4_·C_6_F_4_I_2_, are connected *via* an N⋯I [2.959 (4) Å] halogen bond, in which the N atom is part of the relatively electron-rich pyrazine ring. The C_6_F_4_I_2_ mol­ecule is almost planar [r.m.s. deviation = 0.038 Å] but there are significant twists in the pyrazine derivative, as seen in the dihedral angles [31.3 (2) and 54.6 (2)°] formed between the pendant pyridyl rings and the central pyrazine ring. The bimolecular aggregates are sustained in the crystal by C—H⋯F and π–π inter­actions [ring centroid(pyrid­yl)–ring centroid(benzene) = 3.678 (3) Å].

## Related literature

For related studies on co-crystal formation, see: Broker & Tiekink (2007 ▶); Broker *et al.* (2008 ▶); Arman *et al.* (2010 ▶). For background to halogen bonding, see: Metrangolo *et al.* (2008 ▶); Pennington *et al.* (2008 ▶).
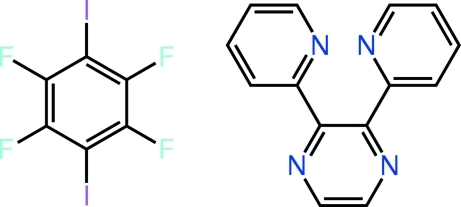


## Experimental

### 

#### Crystal data

C_14_H_10_N_4_·C_6_F_4_I_2_
*M* *_r_* = 636.04
Triclinic, 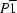

*a* = 6.3997 (15) Å
*b* = 10.737 (2) Å
*c* = 15.092 (4) Å
α = 74.237 (10)°
β = 85.877 (11)°
γ = 80.283 (12)°
*V* = 983.3 (4) Å^3^
*Z* = 2
Mo *K*α radiation
μ = 3.25 mm^−1^
*T* = 98 K
0.40 × 0.13 × 0.07 mm

#### Data collection

Rigaku AFC12/SATURN724 diffractometer
Absorption correction: multi-scan (*ABSCOR*; Higashi, 1995 ▶) *T*_min_ = 0.504, *T*_max_ = 1.000
5074 measured reflections
3775 independent reflections
3550 reflections with *I* > 2σ(*I*)
*R*_int_ = 0.025

#### Refinement

*R*[*F* ^2^ > 2σ(*F* ^2^)] = 0.039
*wR*(*F* ^2^) = 0.109
*S* = 1.09
3775 reflections
271 parameters
H-atom parameters constrained
Δρ_max_ = 1.32 e Å^−3^
Δρ_min_ = −1.20 e Å^−3^

### 

Data collection: *CrystalClear* (Molecular Structure Corporation & Rigaku, 2005 ▶); cell refinement: *CrystalClear*; data reduction: *CrystalClear*; program(s) used to solve structure: *SHELXS97* (Sheldrick, 2008 ▶); program(s) used to refine structure: *SHELXL97* (Sheldrick, 2008 ▶); molecular graphics: *ORTEP-3* (Farrugia, 1997 ▶) and *DIAMOND* (Brandenburg, 2006 ▶); software used to prepare material for publication: *publCIF* (Westrip, 2010 ▶).

## Supplementary Material

Crystal structure: contains datablocks general, I. DOI: 10.1107/S1600536810041668/hb5683sup1.cif

Structure factors: contains datablocks I. DOI: 10.1107/S1600536810041668/hb5683Isup2.hkl

Additional supplementary materials:  crystallographic information; 3D view; checkCIF report

## Figures and Tables

**Table 1:** Hydrogen-bond geometry (Å, °)

*D*—H⋯*A*	*D*—H	H⋯*A*	*D*⋯*A*	*D*—H⋯*A*
C8—H8⋯F4	0.95	2.54	3.145 (6)	121
C9—H9⋯F4	0.95	2.46	3.100 (6)	125
C18—H18⋯F2^i^	0.95	2.52	3.341 (7)	144

Symmetry code: (i) 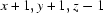.

**Fig. 1.**
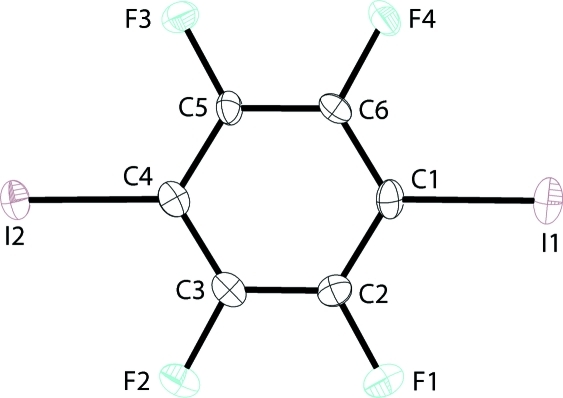
Molecular structure of 1,2,4,5-tetrafluoro-3,6-diiodobenzene found in the structure of (I) showing displacement ellipsoids at the 50% probability level

**Fig. 2.**
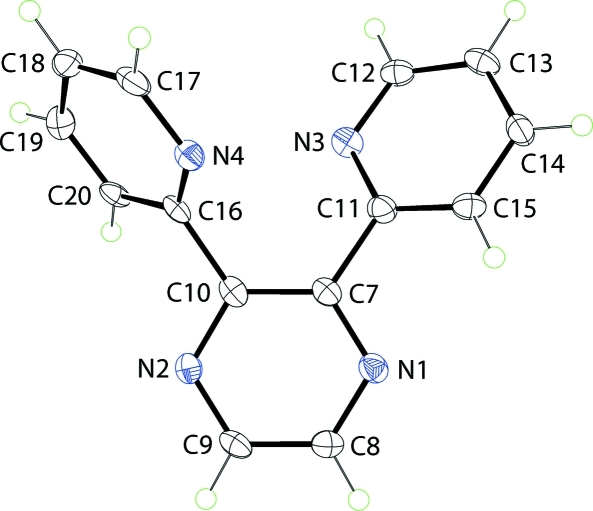
Molecular structure of 2,3-bis(pyridin-2-yl)pyrazine found in the structure of (I) showing displacement ellipsoids at the 50% probability level.

**Fig. 3.**
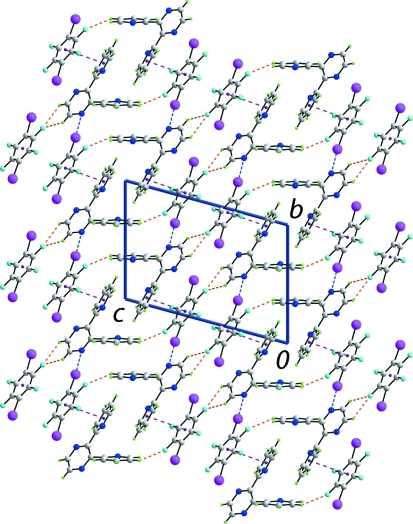
A view in projection down the a axis showing the unit-cell contents. The N···I, C—H···F and π···π interactions are shown as blue, orange and purple dashed lines, respectively.

## supplementary crystallographic information

### Comment

The title co-crystal was prepared during on-going studies investigating co-crystals with pyridine-type molecules (Broker & Tiekink, 2007; Broker *et al.*, 2008) including halogen bonding (Arman *et al.*, 2010). The co-crystallization experiment whereby equimolar amounts of 1,2,4,5-tetrafluoro-3,6-diiodobenzene and 2,3-bis(pyridin-2-yl)pyrazine were dissolved in methylene chloride resulted in the isolation of the title 1/1 co-crystal, (I).

The molecule of 1,2,4,5-tetrafluoro-3,6-diiodobenzene, Fig. 1, is flat with the r.m.s. deviation of the 12 constituent atoms being 0.038 Å [maximum deviation = 0.084 (1) Å for atom I2]. In the molecule of 2,3-bis(pyridin-2-yl)pyrazine, Fig. 2, the N3- and N4-pyridyl rings form dihedral angles of 31.3 (2) and 54.6 (2) ° with the pyrazine ring; the dihedral angle formed between the pyridyl rings = 60.2 (2) °.

The primary connection between the constituent molecules of (I) is a N2···I2 contact of 2.959 (4) Å, representing an halogen bond (Metrangolo *et al.*, 2008; Pennington *et al.*, 2008). Of note is the observation that the interaction involves a pyrazine-N rather than a pyridine-N, consistent with the N in the pyrazine ring being more electron rich. The molecules are stabilized in the crystal packing *via* a combination of C—H···F [the F4 atom is bifurcated], Table 1, and π···π interactions [ring centroid(N3,C11–C15)···ring centroid(C1–C6)^i^ = 3.678 (3) Å for *i*: 1 - *x*, 1 - *y*, 1 - *z*], Fig. 3.

### Experimental

Initially 1,2,4,5-tetrafluoro-3,6-diiodobenzene (Aldrich, 0.09 mmol) and 2,3-bis(pyridin-2-yl)pyrazine (Aldrich, 0.04 mmol) were dissolved in chloroform and after evaporation of the solvent, the powder was then dissolved in tetrahydrafuran (THF). Upon evaporation of THF, methylene chloride was added to the powder. Colourless prisms of (I) were formed after three days through slow evaporation of solvent, m. pt. 409–411 K.

### Refinement

C-bound H-atoms were placed in calculated positions (C–H 0.95 Å) and were included in the refinement in the riding model approximation with *U*_iso_(H) set to 1.2*U*_eq_(C). The maximum and minimum residual electron density peaks of 1.32 and 1.20 e Å^-3^, respectively, were located 0.62 Å and 0.86 Å from the H13 and I1 atoms, respectively.

### Crystal data

**Table:**

C_14_H_10_N_4_·C_6_F_4_I_2_	*Z* = 2
*M**_r_* = 636.04	*F*(000) = 600
Triclinic, *P*1	*D*_x_ = 2.148 Mg m^−^^3^
Hall symbol: -P 1	Mo *K*α radiation, λ = 0.71073 Å
*a* = 6.3997 (15) Å	Cell parameters from 2870 reflections
*b* = 10.737 (2) Å	θ = 2.8–40.2°
*c* = 15.092 (4) Å	µ = 3.25 mm^−^^1^
α = 74.237 (10)°	*T* = 98 K
β = 85.877 (11)°	Prism, colourless
γ = 80.283 (12)°	0.40 × 0.13 × 0.07 mm
*V* = 983.3 (4) Å^3^	

### Data collection

**Table:**

Rigaku AFC12K/SATURN724 diffractometer	3775 independent reflections
Radiation source: fine-focus sealed tube	3550 reflections with *I* > 2σ(*I*)
graphite	*R*_int_ = 0.025
ω scans	θ_max_ = 26.0°, θ_min_ = 2.7°
Absorption correction: multi-scan (*ABSCOR*; Higashi, 1995)	*h* = −7→7
*T*_min_ = 0.504, *T*_max_ = 1.000	*k* = −13→13
5074 measured reflections	*l* = −18→18

### Refinement

**Table:**

Refinement on *F*^2^	Primary atom site location: structure-invariant direct methods
Least-squares matrix: full	Secondary atom site location: difference Fourier map
*R*[*F*^2^ > 2σ(*F*^2^)] = 0.039	Hydrogen site location: inferred from neighbouring sites
*wR*(*F*^2^) = 0.109	H-atom parameters constrained
*S* = 1.09	*w* = 1/[σ^2^(*F*_o_^2^) + (0.0647*P*)^2^ + 2.2885*P*] where *P* = (*F*_o_^2^ + 2*F*_c_^2^)/3
3775 reflections	(Δ/σ)_max_ = 0.001
271 parameters	Δρ_max_ = 1.32 e Å^−^^3^
0 restraints	Δρ_min_ = −1.20 e Å^−^^3^

### Special details

**Table:**

Geometry. All s.u.'s (except the s.u. in the dihedral angle between two l.s. planes) are estimated using the full covariance matrix. The cell s.u.'s are taken into account individually in the estimation of s.u.'s in distances, angles and torsion angles; correlations between s.u.'s in cell parameters are only used when they are defined by crystal symmetry. An approximate (isotropic) treatment of cell s.u.'s is used for estimating s.u.'s involving l.s. planes.
Refinement. Refinement of *F*^2^ against ALL reflections. The weighted *R*-factor *wR* and goodness of fit *S* are based on *F*^2^, conventional *R*-factors *R* are based on *F*, with *F* set to zero for negative *F*^2^. The threshold expression of *F*^2^ > 2σ(*F*^2^) is used only for calculating *R*-factors(gt) *etc*. and is not relevant to the choice of reflections for refinement. *R*-factors based on *F*^2^ are statistically about twice as large as those based on *F*, and *R*- factors based on ALL data will be even larger.

### Fractional atomic coordinates and isotropic or equivalent isotropic displacement parameters (Å^2^)

**Table:**

	*x*	*y*	*z*	*U*_iso_*/*U*_eq_	
I1	0.02819 (5)	0.31600 (3)	0.57773 (2)	0.02562 (13)	
I2	0.78603 (5)	−0.24672 (3)	0.69360 (2)	0.02055 (12)	
F1	0.0119 (4)	0.0318 (3)	0.7123 (2)	0.0253 (6)	
F2	0.2992 (5)	−0.1842 (3)	0.7543 (2)	0.0243 (6)	
F3	0.7961 (5)	0.0349 (3)	0.5483 (2)	0.0237 (6)	
F4	0.5096 (5)	0.2523 (3)	0.5082 (2)	0.0235 (6)	
C1	0.2526 (8)	0.1487 (5)	0.6091 (3)	0.0187 (10)	
C2	0.2048 (7)	0.0342 (5)	0.6718 (3)	0.0183 (9)	
C3	0.3548 (8)	−0.0770 (5)	0.6931 (3)	0.0185 (9)	
C4	0.5573 (8)	−0.0806 (5)	0.6541 (3)	0.0184 (9)	
C5	0.6036 (7)	0.0330 (4)	0.5907 (3)	0.0168 (9)	
C6	0.4545 (8)	0.1452 (5)	0.5692 (3)	0.0168 (9)	
N1	0.4973 (6)	0.6357 (4)	0.3011 (3)	0.0207 (8)	
N2	0.8530 (6)	0.4641 (4)	0.2683 (3)	0.0193 (8)	
N3	0.7532 (6)	0.8733 (4)	0.1407 (3)	0.0208 (8)	
N4	0.8477 (6)	0.6513 (4)	0.0459 (3)	0.0191 (8)	
C7	0.6336 (8)	0.6725 (4)	0.2310 (3)	0.0181 (9)	
C8	0.5401 (8)	0.5140 (5)	0.3545 (3)	0.0214 (10)	
H8	0.4440	0.4842	0.4036	0.026*	
C9	0.7207 (8)	0.4295 (4)	0.3405 (3)	0.0215 (10)	
H9	0.7514	0.3457	0.3828	0.026*	
C10	0.8064 (7)	0.5845 (4)	0.2107 (3)	0.0178 (9)	
C11	0.5864 (8)	0.8134 (5)	0.1779 (3)	0.0205 (10)	
C12	0.7097 (8)	1.0002 (5)	0.0970 (3)	0.0213 (10)	
H12	0.8255	1.0442	0.0709	0.026*	
C13	0.5087 (8)	1.0721 (5)	0.0870 (3)	0.0223 (10)	
H13	0.4873	1.1624	0.0550	0.027*	
C14	0.3393 (8)	1.0090 (5)	0.1249 (3)	0.0226 (10)	
H14	0.1985	1.0552	0.1192	0.027*	
C15	0.3776 (8)	0.8781 (5)	0.1710 (3)	0.0205 (10)	
H15	0.2638	0.8327	0.1977	0.025*	
C16	0.9470 (8)	0.6132 (4)	0.1261 (3)	0.0189 (9)	
C17	0.9704 (9)	0.6689 (5)	−0.0306 (4)	0.0244 (10)	
H17	0.9038	0.6948	−0.0884	0.029*	
C18	1.1934 (8)	0.6509 (5)	−0.0298 (4)	0.0241 (10)	
H18	1.2751	0.6654	−0.0857	0.029*	
C19	1.2907 (8)	0.6117 (5)	0.0544 (4)	0.0242 (10)	
H19	1.4409	0.5979	0.0573	0.029*	
C20	1.1658 (7)	0.5928 (4)	0.1341 (3)	0.0191 (10)	
H20	1.2281	0.5665	0.1929	0.023*	

### Atomic displacement parameters (Å^2^)

**Table:**

	*U*^11^	*U*^22^	*U*^33^	*U*^12^	*U*^13^	*U*^23^
I1	0.0227 (2)	0.0220 (2)	0.0325 (2)	0.00505 (13)	−0.00955 (14)	−0.01061 (15)
I2	0.02250 (19)	0.01648 (18)	0.02206 (19)	0.00093 (12)	−0.00667 (13)	−0.00489 (13)
F1	0.0179 (14)	0.0322 (16)	0.0267 (16)	−0.0065 (12)	0.0029 (12)	−0.0084 (13)
F2	0.0296 (15)	0.0220 (14)	0.0202 (15)	−0.0091 (12)	−0.0007 (12)	−0.0006 (12)
F3	0.0172 (13)	0.0283 (16)	0.0228 (15)	−0.0035 (12)	0.0027 (11)	−0.0031 (12)
F4	0.0250 (15)	0.0171 (14)	0.0234 (15)	−0.0021 (11)	−0.0037 (12)	0.0032 (11)
C1	0.017 (2)	0.018 (2)	0.021 (2)	0.0012 (18)	−0.0094 (18)	−0.0060 (19)
C2	0.018 (2)	0.021 (2)	0.018 (2)	−0.0044 (18)	0.0010 (18)	−0.0076 (18)
C3	0.025 (2)	0.016 (2)	0.015 (2)	−0.0054 (18)	−0.0046 (18)	−0.0036 (17)
C4	0.023 (2)	0.016 (2)	0.016 (2)	−0.0024 (18)	−0.0045 (18)	−0.0042 (18)
C5	0.017 (2)	0.016 (2)	0.015 (2)	0.0020 (17)	−0.0042 (17)	−0.0019 (17)
C6	0.022 (2)	0.015 (2)	0.012 (2)	−0.0050 (18)	−0.0021 (18)	−0.0007 (17)
N1	0.0182 (19)	0.0185 (19)	0.024 (2)	−0.0023 (15)	−0.0028 (16)	−0.0035 (16)
N2	0.0173 (19)	0.0190 (19)	0.021 (2)	−0.0005 (15)	−0.0022 (16)	−0.0060 (16)
N3	0.022 (2)	0.020 (2)	0.022 (2)	−0.0056 (16)	−0.0040 (16)	−0.0063 (16)
N4	0.024 (2)	0.0169 (19)	0.017 (2)	−0.0011 (15)	−0.0036 (16)	−0.0062 (15)
C7	0.022 (2)	0.016 (2)	0.016 (2)	−0.0019 (18)	−0.0047 (18)	−0.0044 (18)
C8	0.023 (2)	0.021 (2)	0.019 (2)	−0.0053 (19)	−0.0006 (19)	−0.0020 (18)
C9	0.030 (3)	0.012 (2)	0.022 (2)	−0.0043 (19)	−0.004 (2)	−0.0030 (18)
C10	0.022 (2)	0.015 (2)	0.018 (2)	−0.0013 (17)	−0.0074 (18)	−0.0069 (17)
C11	0.026 (2)	0.016 (2)	0.020 (2)	−0.0035 (18)	0.0012 (19)	−0.0052 (18)
C12	0.027 (2)	0.020 (2)	0.019 (2)	−0.0094 (19)	0.0001 (19)	−0.0038 (18)
C13	0.033 (3)	0.014 (2)	0.020 (2)	−0.0060 (19)	−0.006 (2)	−0.0026 (18)
C14	0.025 (2)	0.018 (2)	0.022 (2)	0.0032 (19)	−0.003 (2)	−0.0050 (19)
C15	0.022 (2)	0.020 (2)	0.020 (2)	−0.0082 (19)	−0.0005 (19)	−0.0024 (19)
C16	0.024 (2)	0.0089 (19)	0.023 (2)	−0.0007 (17)	−0.0031 (19)	−0.0027 (17)
C17	0.037 (3)	0.014 (2)	0.019 (2)	0.000 (2)	−0.006 (2)	−0.0005 (18)
C18	0.031 (3)	0.017 (2)	0.023 (3)	−0.0058 (19)	0.007 (2)	−0.0056 (19)
C19	0.021 (2)	0.023 (2)	0.029 (3)	−0.0014 (19)	−0.001 (2)	−0.009 (2)
C20	0.016 (2)	0.014 (2)	0.023 (2)	−0.0018 (17)	−0.0075 (18)	0.0032 (18)

### Geometric parameters (Å, °)

**Table:**

I1—C1	2.068 (5)	C7—C11	1.497 (6)
I2—C4	2.085 (5)	C8—C9	1.385 (7)
F1—C2	1.339 (5)	C8—H8	0.9500
F2—C3	1.348 (5)	C9—H9	0.9500
F3—C5	1.348 (5)	C10—C16	1.500 (7)
F4—C6	1.346 (5)	C11—C15	1.395 (7)
C1—C6	1.385 (7)	C12—C13	1.380 (7)
C1—C2	1.398 (7)	C12—H12	0.9500
C2—C3	1.378 (7)	C13—C14	1.382 (7)
C3—C4	1.383 (7)	C13—H13	0.9500
C4—C5	1.392 (7)	C14—C15	1.377 (7)
C5—C6	1.382 (6)	C14—H14	0.9500
N1—C8	1.331 (6)	C15—H15	0.9500
N1—C7	1.340 (6)	C16—C20	1.389 (6)
N2—C9	1.340 (7)	C17—C18	1.408 (7)
N2—C10	1.345 (6)	C17—H17	0.9500
N3—C12	1.333 (6)	C18—C19	1.383 (7)
N3—C11	1.349 (6)	C18—H18	0.9500
N4—C16	1.338 (6)	C19—C20	1.383 (7)
N4—C17	1.337 (7)	C19—H19	0.9500
C7—C10	1.402 (7)	C20—H20	0.9500
			
C6—C1—C2	117.4 (4)	N2—C10—C16	115.6 (4)
C6—C1—I1	121.8 (4)	C7—C10—C16	124.4 (4)
C2—C1—I1	120.8 (4)	N3—C11—C15	122.8 (4)
F1—C2—C3	119.2 (4)	N3—C11—C7	117.1 (4)
F1—C2—C1	120.0 (4)	C15—C11—C7	120.1 (4)
C3—C2—C1	120.8 (4)	N3—C12—C13	124.7 (4)
F2—C3—C2	117.9 (4)	N3—C12—H12	117.6
F2—C3—C4	120.1 (4)	C13—C12—H12	117.6
C2—C3—C4	122.0 (4)	C14—C13—C12	118.1 (4)
C3—C4—C5	117.1 (4)	C14—C13—H13	121.0
C3—C4—I2	121.1 (4)	C12—C13—H13	121.0
C5—C4—I2	121.7 (4)	C13—C14—C15	119.0 (5)
F3—C5—C6	118.6 (4)	C13—C14—H14	120.5
F3—C5—C4	120.1 (4)	C15—C14—H14	120.5
C6—C5—C4	121.3 (4)	C14—C15—C11	118.9 (4)
F4—C6—C5	118.5 (4)	C14—C15—H15	120.5
F4—C6—C1	120.2 (4)	C11—C15—H15	120.5
C5—C6—C1	121.4 (4)	N4—C16—C20	124.3 (5)
C8—N1—C7	117.1 (4)	N4—C16—C10	115.5 (4)
C9—N2—C10	117.8 (4)	C20—C16—C10	120.0 (4)
C12—N3—C11	116.5 (4)	N4—C17—C18	123.3 (5)
C16—N4—C17	116.7 (4)	N4—C17—H17	118.4
N1—C7—C10	121.6 (4)	C18—C17—H17	118.4
N1—C7—C11	114.6 (4)	C19—C18—C17	118.4 (5)
C10—C7—C11	123.7 (4)	C19—C18—H18	120.8
N1—C8—C9	121.9 (4)	C17—C18—H18	120.8
N1—C8—H8	119.0	C18—C19—C20	118.9 (5)
C9—C8—H8	119.0	C18—C19—H19	120.5
N2—C9—C8	121.1 (4)	C20—C19—H19	120.5
N2—C9—H9	119.5	C16—C20—C19	118.3 (5)
C8—C9—H9	119.5	C16—C20—H20	120.8
N2—C10—C7	120.0 (4)	C19—C20—H20	120.8
			
C6—C1—C2—F1	−179.7 (4)	C9—N2—C10—C16	−174.1 (4)
I1—C1—C2—F1	0.1 (6)	N1—C7—C10—N2	−7.7 (7)
C6—C1—C2—C3	0.6 (7)	C11—C7—C10—N2	171.8 (4)
I1—C1—C2—C3	−179.6 (4)	N1—C7—C10—C16	171.1 (4)
F1—C2—C3—F2	0.1 (7)	C11—C7—C10—C16	−9.4 (7)
C1—C2—C3—F2	179.8 (4)	C12—N3—C11—C15	0.7 (7)
F1—C2—C3—C4	−179.6 (4)	C12—N3—C11—C7	−177.3 (4)
C1—C2—C3—C4	0.1 (7)	N1—C7—C11—N3	148.1 (4)
F2—C3—C4—C5	179.3 (4)	C10—C7—C11—N3	−31.5 (7)
C2—C3—C4—C5	−1.0 (7)	N1—C7—C11—C15	−29.9 (7)
F2—C3—C4—I2	−3.4 (6)	C10—C7—C11—C15	150.5 (5)
C2—C3—C4—I2	176.3 (4)	C11—N3—C12—C13	−0.5 (7)
C3—C4—C5—F3	−178.1 (4)	N3—C12—C13—C14	0.0 (8)
I2—C4—C5—F3	4.6 (6)	C12—C13—C14—C15	0.3 (7)
C3—C4—C5—C6	1.2 (7)	C13—C14—C15—C11	−0.1 (7)
I2—C4—C5—C6	−176.1 (3)	N3—C11—C15—C14	−0.4 (8)
F3—C5—C6—F4	−1.4 (6)	C7—C11—C15—C14	177.5 (5)
C4—C5—C6—F4	179.2 (4)	C17—N4—C16—C20	0.8 (7)
F3—C5—C6—C1	178.8 (4)	C17—N4—C16—C10	−175.5 (4)
C4—C5—C6—C1	−0.6 (7)	N2—C10—C16—N4	124.0 (4)
C2—C1—C6—F4	179.9 (4)	C7—C10—C16—N4	−54.8 (6)
I1—C1—C6—F4	0.0 (6)	N2—C10—C16—C20	−52.5 (6)
C2—C1—C6—C5	−0.3 (7)	C7—C10—C16—C20	128.7 (5)
I1—C1—C6—C5	179.9 (4)	C16—N4—C17—C18	−0.8 (7)
C8—N1—C7—C10	4.1 (7)	N4—C17—C18—C19	0.8 (7)
C8—N1—C7—C11	−175.5 (4)	C17—C18—C19—C20	−0.6 (7)
C7—N1—C8—C9	2.0 (7)	N4—C16—C20—C19	−0.6 (7)
C10—N2—C9—C8	1.1 (7)	C10—C16—C20—C19	175.5 (4)
N1—C8—C9—N2	−4.8 (8)	C18—C19—C20—C16	0.5 (7)
C9—N2—C10—C7	4.8 (7)		

### Hydrogen-bond geometry (Å, °)

**Table:**

*D*—H···*A*	*D*—H	H···*A*	*D*···*A*	*D*—H···*A*
C8—H8···F4	0.95	2.54	3.145 (6)	121
C9—H9···F4	0.95	2.46	3.100 (6)	125
C18—H18···F2^i^	0.95	2.52	3.341 (7)	144

Symmetry codes: (i) *x*+1, *y*+1, *z*−1.
